# Elastin-Derived Peptide-Based Hydrogels as a Potential Drug Delivery System

**DOI:** 10.3390/gels10080531

**Published:** 2024-08-12

**Authors:** Othman Al Musaimi, Keng Wooi Ng, Varshitha Gavva, Oscar M. Mercado-Valenzo, Hajira Banu Haroon, Daryl R. Williams

**Affiliations:** 1 School of Pharmacy, Newcastle University, Newcastle upon Tyne NE1 7RU, UK; keng.ng@newcastle.ac.uk (K.W.N.); hajira-banu.h@newcastle.ac.uk (H.B.H.); 2 Department of Chemical Engineering, Imperial College London, London SW7 2AZ, UK; varshithagavva9@gmail.com (V.G.); o.mercado-valenzo20@imperial.ac.uk (O.M.M.-V.); d.r.williams@imperial.ac.uk (D.R.W.)

**Keywords:** peptides, elastin, self-assembly, hydrogel, drug delivery

## Abstract

A peptide-based hydrogel sequence was computationally predicted from the Ala-rich cross-linked domains of elastin. Three candidate peptides were subsequently synthesised and characterised as potential drug delivery vehicles. The elastin-derived peptides are Fmoc-FFAAAAKAA-NH_2_, Fmoc-FFAAAKAA-NH_2_, and Fmoc-FFAAAKAAA-NH_2_. All three peptide sequences were able to self-assemble into nanofibers. However, only the first two could form hydrogels, which are preferred as delivery systems compared to solutions. Both of these peptides also exhibited favourable nanofiber lengths of at least 1.86 and 4.57 µm, respectively, which are beneficial for the successful delivery and stability of drugs. The shorter fibre lengths of the third peptide (maximum 0.649 µm) could have inhibited their self-assembly into the three-dimensional networks crucial to hydrogel formation.

## 1. Introduction

In 2023, global pharmaceutical revenues reached a total of USD 1.6 trillion [1]. Within this expansive market, the UK’s pharmaceutical sector is expected to reach USD 31.3 billion in 2024, representing a substantial and influential segment, accounting for about 2.6% of the global pharmaceutical market [2]. This growth underscores the UK’s important role in driving advancements in healthcare and pharmaceuticals worldwide. The three largest and fastest-growing categories of new biotherapeutic modalities in development are peptides, monoclonal antibodies, and oligonucleotides. Between 2016 and 2023, a total of 31 peptide-based drugs received approval from the United States Food and Drug Administration (FDA) [3]. Peptides are also present in various classes of pharmaceuticals, such as antibody–drug conjugates (ADCs) and peptide–drug conjugates (PDCs), where they can function as linkers, payloads, or both [4]. Their significant impact in engaging with therapeutic targets has made peptides favourable compared to other classes of pharmaceuticals. Interestingly, peptides have also demonstrated the ability to address multiple diseases beyond their original targets. Over the past five years, there has been a rapid expansion in the development of new peptide drugs for diabetes, particularly those that mimic the natural hormone glucagon-like peptide-1 (GLP-1). Notable examples include Trulicity and Ozempic, which have become major drugs for managing type 2 diabetes [5,6]. Recent reports have highlighted the potential future impact of these peptide drugs beyond type 2 diabetes treatment, suggesting that they could become frontline medications for addressing obesity and heart disease [7,8,9]. Due to their outstanding safety profile, biocompatibility, and biodegradability, peptides are considered appealing drug classes and drug carriers and have been incorporated into various medical fields, including cardiology [10], oncology [4], and wound treatment [6].

Hydrogels are the first biomaterials to have been used in biomedical applications [11]. They are composed of distinct 3D structures that swell in water but do not dissolve. Hydrogels can be classified as natural or synthetic biopolymers based on their source, cross-linking nature (covalent or physical), network nature (homopolymer, copolymer, interpenetrating, or double networks), and their biodegradability [12,13,14]. Hydrogels are being used to augment vocal cords [15], prevent the formation of scar tissue after surgery, recover perforated ear drums [16], restore detached retinas [17], and aid in cosmetic and wound dressing applications [18].

Peptides are highly diverse in structure, possessing the ability to self-assemble and form a novel class of synthetic peptide-based hydrogels [19]. Peptides can self-assemble into nanostructures and hydrogels under aqueous conditions, resulting in various assemblies, such as nanospheres [20], fibrous and plate-like structures [21,22], heterogeneous nanostructures [23,24,25], micelles, and nanotubes [26,27,28]. Peptide assemblies exhibit distinctive physicochemical and biochemical activities, which depend on their morphology and size and the accessibility of their reactive surface area [29]. They also show extracellular matrix-mimicking microenvironments, which results in them being used as scaffolds for tissue and cell regeneration applications. For instance, an ultrashort peptide-based hydrogel derived from the C-region of insulin-like growth factor 1 (IGF-1) formed supramolecular nanofibers and was used for the treatment of GC-induced sarcopenia, with a biological activity surpassing that of IGF-1 [30]. Interestingly, peptides naturally possess medicinal attributes and their self-assembly results in the formation of inherently bioactive hydrogels. This is demonstrated by the hydrogel developed by Salick and co-workers, whose peptide exhibits antibacterial activity against both Gram-negative and Gram-positive bacteria without the need for exogenous antimicrobial agents [31]. A fascinating study utilised a peptide-based hydrogel to construct anti-cancer peptides/polyvinyl alcohol (PVA) double-network (DN) hydrogels for the treatment of melanomas [32]. These peptides have already been used previously as antimicrobial agents [32]. Moreover, these hydrogels demonstrate excellent anti-tumour, antibacterial, and wound-healing-promoting abilities in vivo [32]. For further reading on peptide-based hydrogels, readers are referred to a review by Liu and colleagues that compiles self-assembling peptide-based hydrogels used for wound tissue repair [33].

The advantage of self-assembled peptide-based hydrogels is that they allow for drug encapsulation during the self-assembly and subsequent gelation processes. This results in coherent and well-loaded drug–hydrogel formulations, unlike drugs diffusing from an already-formed hydrogel. Furthermore, this strategy makes it easier to accurately determine the exact concentration of the loaded drug. The ability to easily manipulate peptide properties plays a crucial role in enhancing the overall performance as a potential drug delivery system. At a given concentration, reducing the overall positive charge of a peptide results in faster self-assembly and the formation of a more rigid and cohesive hydrogel with increased cross-linking [34]. This possibility will be beneficial in terms of modulating drug release kinetics.

Peptides can be engineered not only to self-assemble but also to engage with active sites of specific enzymes, resulting in biodegradable hydrogels. These diverse capabilities have sparked significant interest in leveraging peptides for drug delivery applications [35,36,37,38,39]. It is important to note that a helical conformation can induce immunogenicity and antigenicity through the production of conformation-specific antibodies [40], particularly when nanomaterials are below 100 nm [41]. This behaviour is ascribed to the harmful interactions that small-sized nanomaterials can have with biological systems, which can result in toxicity. Therefore, addressing this behavioural aspect is crucial and can be effectively managed during both the peptide selection and gelation formation processes.

Elastin-derived peptides have attracted researchers’ attention in preparing hydrogels for various applications. This interest is due to their unique properties, including their tuneable mechanical properties, their high swelling ratios, elasticity, strength, and biocompatibility, the ability to tailor drug loading and control release kinetics, and, most importantly, their enzymatic degradation profiles, which facilitate their use in programmed drug delivery systems [42,43,44,45,46]. Various studies have incorporated elastin-derived materials as potential drug delivery systems, including the delivery of salvianolic acid B for myocardial infarction treatment [47] and the effective delivery of anti-cancer drugs [48] as a targeted drug delivery system for castration-resistant prostate cancer (CRPC) [49].

In this study, we report the design, synthesis, and characterisation of elastin-inspired peptide-based hydrogels as potential candidates to effectively encapsulate and deliver drugs. We envisage that the 3D network of the hydrogel will not only enable the efficient colonisation and vascularisation of functional native soft tissues but will also provide the necessary protection for the drug against enzymatic attack.

## 2. Results and Discussion

Three peptide sequences, computationally derived from elastin protein, were identified as potential drug delivery carriers. These sequences were chemically synthesised and characterised for their suitability in drug delivery applications. The experimental results validated various properties as predicted using computational tools and platforms. The experiments conducted for this purpose are discussed and analysed herein.

### 2.1. Sequence Derivation

**Figure 1:**
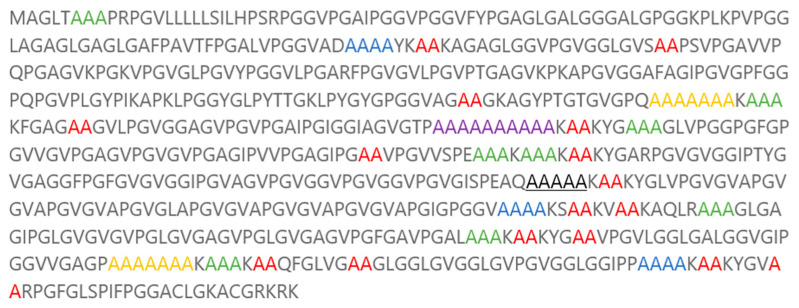
Elastin protein sequence. Colours and underlining represent the abundance of repeated sequences of the Ala-rich cross-linking domain.

Elastin is a major component of tissues in our bodies that require stretchiness or elastic behaviour. Elastin provides the elastic properties essential for vertebrate tissues, offering the tensile strength and passive elastic recoil required for dynamic organs such as blood vessels, the heart, and the lungs (Figure 1) [50,51].

Therefore, the selection of the candidate peptide sequences for the hydrogel has been inspired by this key protein (Table 1).

**Figure 2:**
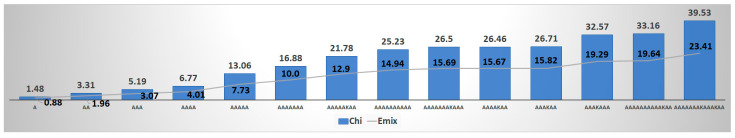
Flory–Huggins interaction parameter Chi (χ) calculated computationally using Materials Studio software (initial screening).

The mixing of hydrogels depends on the mixing enthalpy (E_mix_), characterised by the Flory–Huggins polymer–solvent interaction (χ), and the cohesive force due to the number of cross-links linking the polymer chains to form the 3D network. Since hydrogels contain significant amounts of water, they exist in an equilibrium swollen state where the balance is between the mixing force and the cohesive force. Therefore, initial modelling work considered the interaction of the designed peptide sequences with water. Using the Blends module of Materials Studio software (version 2016, Accelrys Inc., San Diego, CA, USA), which aids in creating a blended product with optimised physical and chemical properties, the selection of the framework sequence was carried out. Using the Blends software module, χ and E_mix_ values were assigned to each peptide sequence. Specifically, the abundant repeated peptide sequences from the Ala-rich cross-linking domain of this protein were determined and computationally screened. This domain is involved in the coacervation process necessary for the final elastin fibre formation, aiding in the alignment and cross-linking of monomers (Figure 2) [50,52,53,54].

Our goal was to identify sequences containing Lysine (Lys), as Lysine is crucial for cross-linking. It plays a key role in the lysyl oxidase-mediated formation of desmosine cross-links, which are necessary for the formation of insoluble elastin [55,56,57]. Furthermore, molecules such as drugs can be anchored through the amine group on the side chain of Lysine. Additionally, this amine group carries a positive charge at physiological pHs, enhancing solubility and facilitating ionic interactions with proposed molecules, thereby boosting the solubility of the final peptide. Therefore, we believe that it is important to include Lysine in our desired candidate sequences.

**Figure 3:**
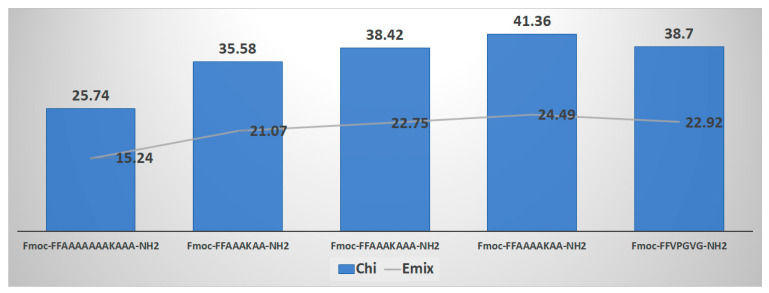
Flory–Huggins interaction parameter Chi (χ) calculated computationally using Materials Studio software versus VPGVG.

To select candidate sequences, we applied the Blends software module to a peptide sequence derived from the hydrophobic domain of the same protein. This sequence, VPGVG, was successfully used to prepare a hydrogel for myocardial infarctions (Figure 3) [47]. Consideration was also given to including fluorenylmethyloxycarbonyl (Fmoc)-FF in our candidate sequence to enhance self-assembly through π-π stacking interactions, promote the formation of nanofibers, and facilitate the transition into a hydrogel [58]. Numerous studies have demonstrated that the FF dipeptide and its derivatives can self-assemble into highly ordered structures, manifesting in various forms with nanoscale order [21,27]. The *C*-terminal has been amidated to maintain neutrality for future conjugation purposes.

The peptide sequences EDP-1 (Fmoc-FFAAAAKAA-NH_2_), EDP-2 (Fmoc-FFAAAKAA-NH_2_), and EDP-3 (Fmoc-FFAAAKAAA-NH_2_) showed similar χ and E_mix_ values to the reference peptide (VPGVG). Therefore, these sequences were selected for further experiments.

### 2.2. I-TASSER

The three peptides selected were evaluated by I-TASSER software (version 5.1) for protein structure prediction [59,60,61]. Since the software requires a sequence length of 10 residues and does not recognise the Fmoc group, we incorporated an additional phenylalanine (F) group to meet the ten-residue requirement and to represent the Fmoc group. Therefore, the following sequences were submitted:

EDP-1, FFFAAAAKAA

EDP-2, FFFFAAAKAA

EDP-3, FFFAAAKAAA

**Figure 4:**
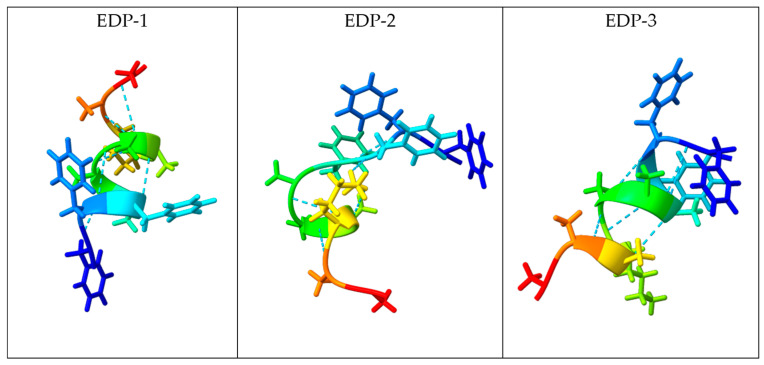
Predicted molecular structures of EDP-1, EDP-2, and EDP-3 (along with H-bond distance 0.400 Å) using I-TASSER [59,60,61].

The software analysis results showed that EDP-1 adopted an α-helical secondary structure, with residues 2 to 7 participating in the α-helix, while residues 1 and 8–10 adopted a coil structure (Figure S1). EDP-2 adopted a β-sheet structure, with residues 2 to 6 participating in the β-sheet secondary structure, while residues 1 and 7–10 adopted a coil structure (Figure S2). EDP-3 adopted an α-helical secondary structure, with residues 3 to 7 forming the α-helix, residues 1 and 2 adopting a coil conformation, and residues 8 to 10 also assuming a coil structure (Figure S3 and Figure 4).

As observed in structures EDP-1 and EDP-3, two helices associate through the hydrophobic interface between residues 2 through 5 and 7 to 9, which are orientated outward. Residues 1, 6, and 10 contribute to the overall stability of the α-helical structure. This arrangement is a significant feature of α-helices, allowing functional groups within the helix to engage in crucial intermolecular interactions.

In conclusion, the secondary structure predicted for EDP-1 and EDP-3 corresponds to a typical α-helical structure known as a 7/2 repeat, where seven amino acids span two helical turns.

### 2.3. MolProbity

MolProbity 4.5.2 is a modelling tool from Duke University that was used to evaluate the secondary structure of the three peptides [62]. The dihedral angles (Φ and Ψ) of the central N-Cα bond in the amino acids involved in the secondary structure were calculated for the three peptides. In EDP-1 and EDP-3, it was confirmed that residues 2 to 9 participate in the helical structure, with Φ values ranging between −50 and −80 and Ψ values ranging between −25 and −60. However, deviations were observed: in EDP-1, residue 7 had an out-of-range Ψ value, and in both EDP-1 and EDP-3, residues 8 and 9 showed out-of-range Φ values. Furthermore, the calculated Rama-Z (Ramachandran plot Z-score) was less than 2 for all residues involved in the helical structure of both peptides. Despite the deviations noted in specific residues, these values predominantly fell within the well-populated region in the lower left quadrant of the Ramachandran plot, indicative of an ideal α-helix conformation for EDP-1 and EDP-3.

In EDP-2, the analysis confirmed the formation of a loop involving residues 1 and 2, while the remaining residues adopted a helical structure. However, Φ and Ψ values exhibited significant variability and often deviated from the ideal α-helix conformation range. This variability may be attributed to intramolecular interactions resulting from the loop conformation adopted by residues 1 and 2. Overall, the observed Φ and Ψ values, along with the Rama-Z scores, confirm the ideal α-helical conformation of EDP-1 and EDP-3, while highlighting the unique structural features of EDP-2 [14] (Supplemental Tables S1–S3).

### 2.4. Peptide Synthesis

**Figure 5:**
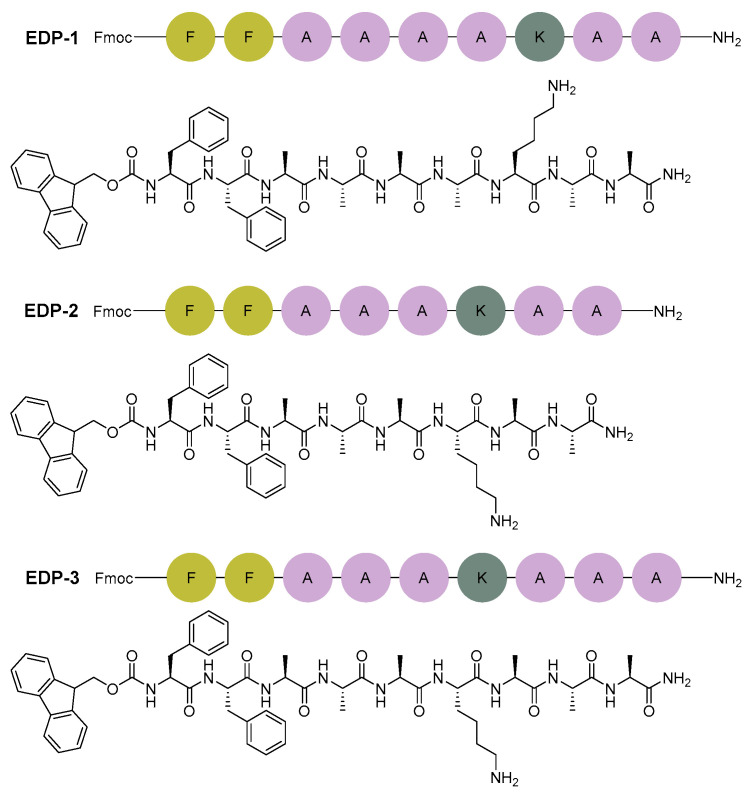
Chemical structure of the three peptide sequences investigated in this work.

The three sequences were synthesised using a microwave-assisted automatic synthesiser (CEM) (Figure 5). Peptides were analysed by HPLC and were confirmed by LC-MS to have good purity: EDP-1 and EDP-2 showed purity of 98.2%, while EDP-3 exhibited 91.8% purity (Figures S4–S9).

### 2.5. Critical Aggregation Concentration (CAC) Determination

**Figure 6:**
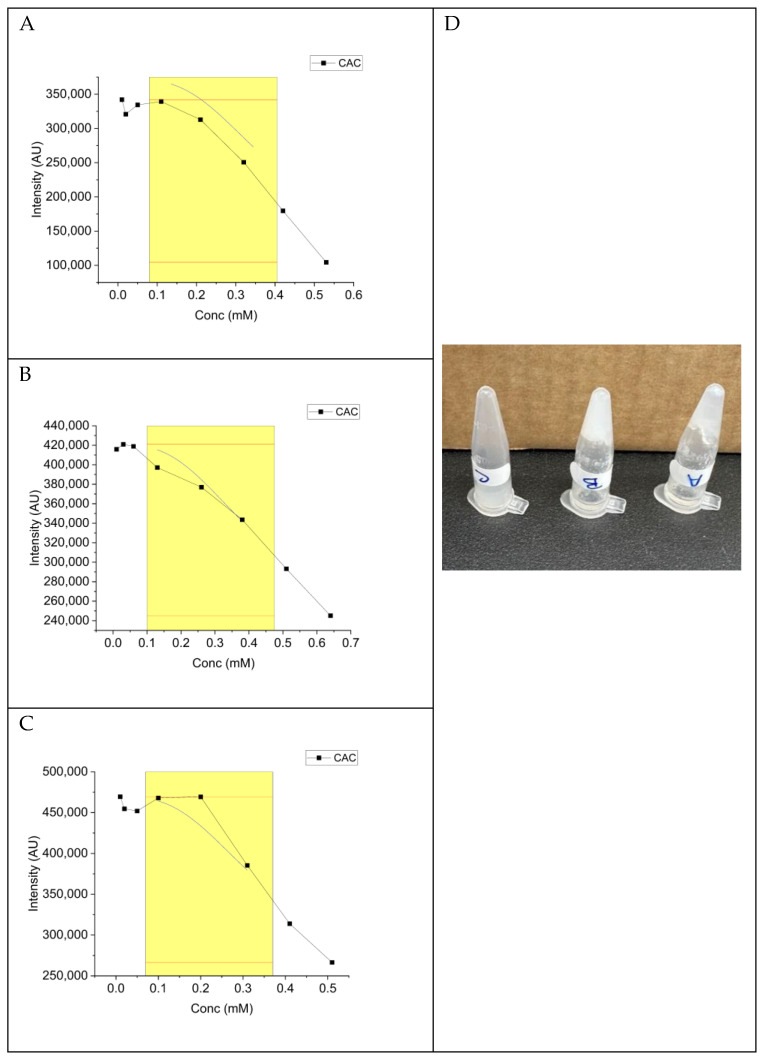
Critical aggregation concentration (CAC) of the selected peptides (**left**). Image of the three peptides to show the hydrogel formation (**right**). (**A**) EDP-1, (**B**) EDP-2, (**C**) EDP-3. (**D**) Digital images of the formed EDP-1 and EDP-2 hydrogels. Sigmoidal function fitted within the yellow region, where the red tangent shows the CAC.

A range of concentrations of the selected peptides were prepared in buffered-CH_3_CN (1:1) and analysed using a fluorescence spectrometer at concentrations of 0.4, 0.3, 0.2, 0.1, 0.05, 0.02, and 0.01 mM. Purine was added to all samples at a final concentration of 1 µM. The critical aggregation concentration (CAC) for all three peptides was determined to be 0.16 mM (Figure 6).

Despite all three peptides sharing the same CAC value of 0.16 mM, only peptides EDP-1 and EDP-2 formed hydrogels within 1 h, with EDP-2 producing a more cohesive hydrogel than EDP-1 (Figure 6D). The short fibres of EDP-3 (maximum 0.649 µm) could explain its inability to efficiently self-assemble into the three-dimensional (3D) network necessary for hydrogel formation (Figure 6D). Interestingly, due to the long nanofibers, our peptides are not expected to induce immunogenicity or antigenicity. Computational data from Figure 3 indicated that the χ and E_mix_ values for EDP-2 were not as high as those for the other two peptides, suggesting a better balance of miscibility between the peptide and water and hence better gelation kinetics.

Injectable hydrogels are more suitable as drug delivery vehicles than solutions. Therefore, peptides EDP-1 and EDP-2 are considered promising candidates for drug formulation. Interestingly, after the gelation process completed within 1 h, excess solvent was expelled from the hydrogel, as confirmed using infrared spectroscopy (IR) (Figure S10). The composition of the expelled solvent was characterised by IR. No traces of the peptide were found in the expelled solvent. Therefore, we can conclude that at the concentration used to dissolve the peptide (1% wt/v), all the peptide quantity has contributed to hydrogel formation. This also confirms the opportunity to incorporate an additional amount of the peptide to achieve a more robust hydrogel structure.

### 2.6. Transmission Electron Microscope (TEM) Imaging

**Figure 7:**
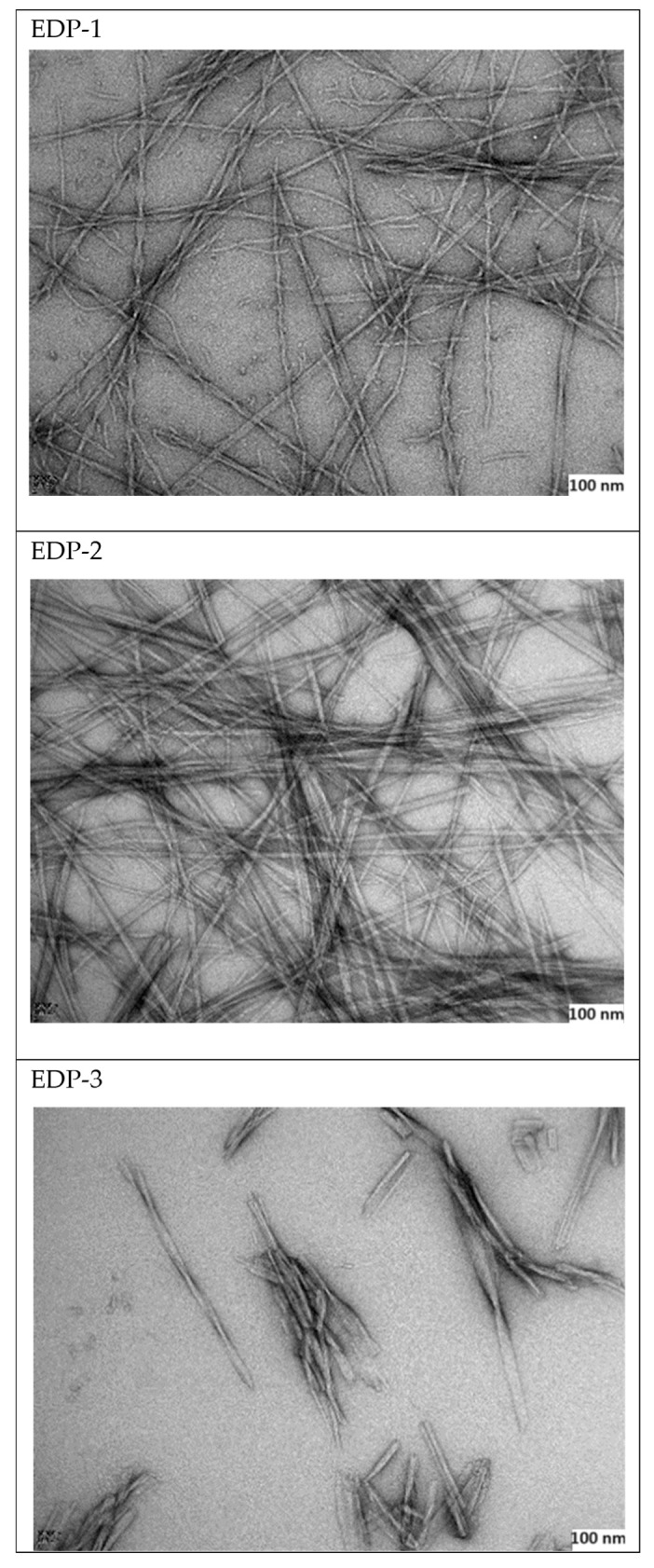
TEM morphology of the three peptides (scale bar= 100 nm).

The three peptides have been examined using transmission electron microscopy analysis (TEM) (Figure 7). As can be visually observed, in addition to the coherent hydrogel that peptide EDP-2 could form, it has also shown better nanofibers than both the investigated peptides.

It is evident that all three peptides formed nanofibers, but their lengths varied: peptide EDP-2 exhibited the longest and densest fibres (at least 4.57 µm), peptide EDP-1 had intermediate fibres (at least 1.86 µm), and peptide EDP-3 had the shortest fibres (no more than 0.649 µm). Interestingly, the obtained TEM images confirm the secondary structures predicted by the computational studies (Section 2.2).

### 2.7. UV Analysis

To investigate the driving force behind the self-assembly processes, UV experiments were conducted. A red shift in the aromatic groups was observed from 288 nm to 303 nm for one peptide and from 299 nm to 335.7 nm for another, confirming the presence of π-π stacking interactions (Figure S11). It is noteworthy that the amino acid compositions of these three peptides are highly prone to self-assembly, which has played a crucial role in the assembly process.

### 2.8. Circular Dichroism (CD) Measurements

**Figure 8:**
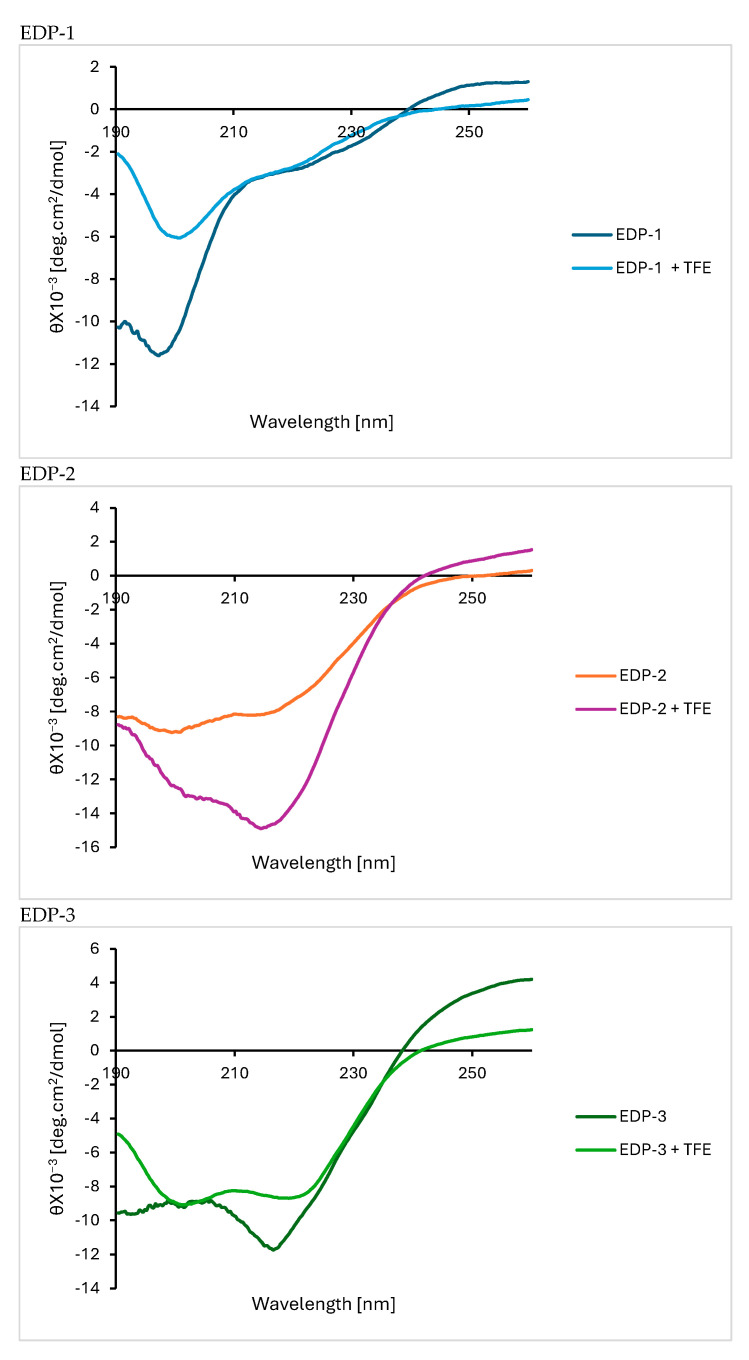
CD spectra for the three peptides investigated in this work.

The three peptides underwent CD analysis to assess their secondary structure [63]. The experiment was conducted in the presence and absence of 40% 2,2,2-trifluoroethanol (TFE) as a secondary structure enhancer [64]. Indeed, a clear enhancement in the spectra was noticed upon the addition of TFE (Figure 8).

The CD data confirmed the computational predictions and assigned the secondary structures for the three peptides designed in this study. EDP-1 and EDP-3 exhibited characteristic CD spectra indicative of α-helix conformation, featuring two negative bands of similar magnitude at 222 and 208 nm, along with a positive band around 190 nm [65]. The α-helical structure was more pronounced in EDP-3 compared to EDP-1, although EDP-1 clearly exhibited structure and was not a random coil. It is important to note that the solvent used can influence these experiments. On the other hand, EDP-2 displayed a negative band between 210 and 220 nm and a positive band between 195 and 200 nm, confirming the adoption of a β-sheet conformation in this case [65].

### 2.9. Dynamic Light Scattering (DLS) and Zeta Measurements

Table 2 summarises the characteristic zeta potential, hydrodynamic size (D_H_, nm), and polydispersity index (PDI) of EDP-1 and -2.

A high absolute zeta potential (positive or negative) aids the dispersion of nanoparticles by electrostatic repulsion. In dispersed systems, a zeta potential between ±10 mV and ±30 mV is associated with incipient instability, while a zeta potential below ±5 mV characterises dispersion instability due to coagulation or flocculation [66]. In the case of EDP-1 and -2, the low zeta potentials indicate weak electrostatic repulsion between the peptide nanofibers, which would allow them to come into proximity and thus facilitate their self-assembly into hydrogels. The hydrodynamic diameters of EDP-1 and EDP-2 were ~3 µm. The high PDI values (0.7–0.9) indicate a heterogenous particle size distribution. This could be due to the ongoing self-assembly of the peptides while running measurements (Figures S12 and S13). It is conceivable that during the DLS measurement, the samples contained a mixture of the peptide nanofibers of different sizes, because they were at various stages of self-assembly.

### 2.10. Storage Modulus (G′) and Loss Modulus (G″)

**Figure 9:**
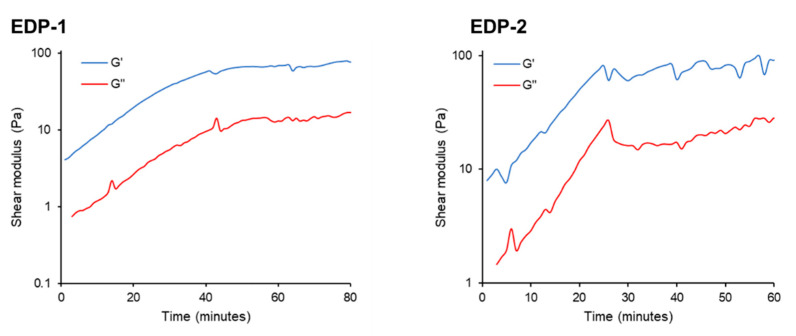
Shear modulus versus time plots for peptides EDP-1 and EDP-2 showing the storage modulus (G′) and loss modules (G″). Peptide solution concentration: 1% (wt/v).

Peptides EDP-1 and EDP-2 exhibited characteristics of a viscoelastic solid (G′ > G″). The gelation process was evident from a sharp increase in both G′ and G″, followed by a plateau, indicating complete gelation (Figure 9). In addition to having higher initial G′ and G″ values, which signify a stronger viscoelastic material, peptide EDP-2 completed gelation sooner (approximately 25 min, with values of 81.33 Pa for G′ and 26.88 Pa for G″) compared to peptide EDP-1 (around 40 min, with values of 57.13 Pa for G′ and 10.08 Pa for G′’) (Supplemental Tables S4 and S5). Interestingly, these findings are consistent with the TEM images obtained (Figure 7). EDP-1 (Figure 7 EDP-1) and EDP-2 (Figure 7 EDP-2) both exhibit long nanofibers, which are believed to contribute to the gelation and mechanical strength of the resulting hydrogels. In contrast, EDP-3 (Figure 7 EDP-3) formed much shorter nanofibers, which did not result in gelation. The nanofibers in EDP-2 were more tightly intertwined than those in EDP-1, likely explaining the greater strength of the hydrogel formed from EDP-2. This behaviour is evident from the higher initial G″ and G′ values and the faster gelation observed.

The rapid gelation of peptide EDP-2 confirms its favourable performance compared to EDP-1. However, since EDP-1 adopts a helical conformation, it is also a strong candidate, particularly compared to peptides that adopt β-sheet structures, such as EDP-2. Therefore, it is anticipated that both peptides have the potential to be efficient vehicles for drug delivery applications, though this hypothesis will need to be evaluated in future work. It is important to note that the initial rheological study was conducted on 1% (wt/v) peptide solution, whereas future investigations will establish the optimum peptide concentration for a robust hydrogel that can then be used for drug loading.

## 3. Experimental Details

### 3.1. Methods and Materials

Rink amide resin (0.5 mmol/g, per the supplier’s specifications) was used for all syntheses. Reagents and solvents were sourced from commercial suppliers and used as received, unless otherwise specified. Analytical HPLC was conducted using a Shimadzu LC20 system with LabSolution software (version 5.92) for data analysis. The LC column used was a Symmetry Luna C_18_ (3.6 μm, 4.6 × 150 mm), with a flow rate of 1.0 mL/min and UV detection at 280 nm. The mobile phase consisted of 0.1% trifluoroacetic acid (TFA) in H_2_O (Phase A) and 0.1% TFA in CH_3_CN (Phase B). LCMS analysis was performed on a Velos Pro mass spectrometer (ThermoFisher Scientific, Waltham, MA, USA), a hybrid linear trap quadrupole (LTQ)-Orbitrap, using positive electrospray ionisation mass spectrometry (ESI+-MS) with direct sample infusion. A Liberty Blue™ automated microwave-assisted peptide synthesiser (CEM) was used for the peptide synthesis. The fluorescence spectrometer used was a Shimadzu-RF6000, Hitachi HT7800 TEM (Tokyo, Japan), using an Emsis Xarosa camera with Radius software (version 2.3). The Fourier transform infrared spectroscopy (FTIR) was performed with an Agilent Cary 630 with the diamond-ATR sampling module. UV-vis spectroscopy was conducted with an Agilent Carry 100 UV-Vis spectrometer. The mean particle size, polydispersity index (PDI), and zeta potential of the particles were determined by dynamic light scattering (DLS) using the Malvern Nanosizer ZS (Model: ZEN3600, Malvern Inc., Malvern, UK) at 25 °C. DTS1070 folded capillary cells were used for zeta potential measurements, and disposable cuvettes (DTS0012) were used for DLS measurements.

### 3.2. Computational Study

Computational studies were carried out using BIOVIA Materials Studio 2018, Accelrys Inc. (San Diego, CA, USA).

All the generated assemblies were energy minimised, and their geometry was optimised using the “Forcite module” with smart algorithms. The forcefield was set to “Dreiding”, and charges were assigned using “Charge using QEq”. Miscibility was simulated with the “Blends module”, maintaining the same forcefield and charge settings as used in the geometry optimisation. The calculation option was set to “mixing”, which performs both binding energy and coordination number calculations to predict the following values: E_mix_, interaction energy, and χ parameter values. The peptide–solvent interaction parameter (χ) and E_mix_ values that were computationally obtained reflect the predicted degree of miscibility between the solutes and solvents. Higher χ and/or E_mix_ values indicate a lower miscibility or swelling capacity.

### 3.3. I-TASSER

The three peptide sequences were inputted into the online platform https://zhanggroup.org/I-TASSER/, and the programme was executed (accessed on 9 August 2024).

### 3.4. MolProbity

The three peptide sequences were inputted into the online platform http://molprobity.biochem.duke.edu/, and the programme was executed (accessed on 9 August 2024).

### 3.5. Peptide Synthesis

Within the automated SPPS method, the protocol provided by CEM corporation was adhered to [67]. The coupling of the amino acid to the growing peptide chain was achieved through the addition and heating of Fmoc-AA-OH acid (0.25 mmol, 5 equiv, 0.2 M in DMF), OxymaPure (0.25 mmol, 5 equiv, 0.5 M in DMF), and DIC (0.50 mmol, 10 equiv, 0.5 M in DMF) at 90 °C for 2 min (single-coupling) or 2 × 2 min (double-coupling). *N*-terminal deprotection of the growing peptide chains was achieved through Fmoc-cleavage via the addition of piperidine (20% *v*/*v* in DMF) in OxymaPure (0.1 M in DMF) and heating at 90 °C for 1.5 min.

### 3.6. Critical Aggregation Concentration (CAC)

Six concentrations of the three peptides were prepared in CH_3_CN -Buffer pH 7.4 (1:1). All levels were examined using fluorescence spectrometry at an excitation wavelength of 374 nm and an emission wavelength of 384 nm.

### 3.7. Hydrogel Formation

Solutions of the three peptides were prepared at a concentration of 1.0% (wt/v) in CH_3_CN-Buffer pH 7.4 (1:1). After 6 hr at room temperature, the solutions were examined using vial inversion to check their ability to form hydrogels.

### 3.8. IR and UV Measurements

The excluded solvent (supernatant) from the hydrogel was analysed by FTIR and compared with fresh solvent. For UV spectroscopy, a spectrum scan was obtained from 200 nm to 400 nm for both a freshly prepared peptide sample and after complete gelation.

### 3.9. TEM Imaging

A 0.2% (wt/v) solution in CH_3_CN–water (1:1) of the three peptides was investigated using TEM. In brief, 10 µL of the sample was settled onto a glow-discharged, copper mesh grid for 30 sec, wicked away, then placed on a droplet of 2% uranyl acetate (UA), then wicked, and then placed on a second droplet of UA. The grid was dried under a lamp. For TEM, the images were collected from different areas of the copper mesh grid.

### 3.10. Circular Dichroism (CD) Measurements

The experiment was conducted on the peptides in CH_3_CN (1:1) at a concentration of 0.3 mg/mL, both in the presence and absence of 40% 2,2,2-trifluoroethanol.

### 3.11. DLS and Zeta Potential

The samples (in CH_3_CN) were prepared by dispersion in McIlvaine buffer (3.63 mM Na_2_HPO_4_, 0.18 mM citric acid and 0.13 mM KCl; pH 7.0). The cells were filled slowly to avoid air bubbles and measured in triplicate. The final concentration of the peptide was 1% (wt/v).

### 3.12. Storage Modulus (G′) and Loss Modulus (G″)

The rheological properties of peptides EDP-1 and EDP-2 were determined by oscillatory rheology on the Kinexus Pro+ rotational rheometer (Malvern Panalytical) using the parallel plate geometry. Freshly reconstituted peptide solutions were loaded immediately on the rheometer. A viscoelastic characterisation was performed with a gap size of 0.5 mm, a strain of 1%, and a frequency of 0.1 Hz to simulate low shear/resting conditions. The storage modulus (G′) and loss modulus (G″) were monitored for up to 180 min to verify time-dependant behaviour.

## 4. Conclusions

In this study, a series of novel peptide-based hydrogels were successfully designed, synthesised, and characterised. Three peptide sequences were derived from the Ala-rich cross-linking domain of the elastin protein. The Ala-rich cross-linking domain is crucial in the coacervation process for the formation of elastin fibres. Experimental data were aligned with computational work to both derive and predict the behaviour of the selected peptides. Among the designed peptides, EDP-1 and EDP-2 showed promising results in forming both nanofibers and robust hydrogels.

Based on these findings, it can be anticipated that these peptide-based hydrogel candidates will be well suited for delivering drugs in a sustained release pattern over an extended period. Implanting external biomaterials such as hydrogels is considered to be ideal for application in tissue regeneration. In Al Musaimi’s group, we are currently working on applying EDP-1 and EDP-2 as a drug delivery system to control the release of a potential peptide-based drug for aesthetic applications. Moreover, it is anticipated that our designed peptide hydrogels will have additional applications not only in soft tissue engineering but also in the field of wound healing.

## Figures and Tables

**Table 1:** Analysis of the elastin protein to establish the abundance of sequences of the Ala-rich cross-linking domain.

Peptide Sequence	Number of Recurrences
AA	16
AAA	8
AAAA	3
AAAAA	1
AAAAAAA	2
AAAAAKAA	1
AAAAAAAAAA	1
AAAAAAAKAAA	2
AAAAKAA	2
AAAKAA	1
AAAKAAA	1
AAAAAAAAAAKAA	1
AAAAAAAKAAAKAA	2

The colours and underlined sequences above represent the abundance of repeated sequences of the Ala-rich cross-linking domain, as per Figure 1.

**Table 2:** Zeta potential and hydrodynamic size of EDP-1 and -2.

Peptide	Zeta Potential (mV)	D_H_ (nm)	PDI
EDP-1	−10.04 ± 1.00	3808.3 ± 1334.8	0.926
EDP-2	−0.74 ± 0.50	3087 ± 1788.0	0.786

## Data Availability

Data are contained within the article or Supplementary Materials.

## Supplementary Materials

The following supporting information can be downloaded at: https://www.mdpi.com/article/10.3390/gels10080531/s1, Figure S1. The normalized B-factor (called B-factor profile, BFP) of EDP-1; Figure S2. The normalized B-factor (called B-factor profile, BFP) of EDP-2; Figure S3. The normalized B-factor (called B-factor profile, BFP) of EDP-3; Figure S4. Mass of Fmoc-FFAAAAKAA-NH_2_. Calculated: 1088.28; found: 1088.98 [M+H]^+^, 545.29 [M+2H]^2+^; Figure S5. Fmoc-FFAAAAKAA-NH_2_. 5−95% in 30 min gradient elution. λ = 280 nm. Mobile phase A: 0.1% TFA in H_2_O; mobile phase B: 0.1% TFA in CH_3_CN; Symmetry Luna C18 (3.6 μm, 4.6 × 150 mm) column. Purity 98.2%; Figure S6. Mass of Fmoc-FFAAAKAA-NH_2_. Calculated: 1017.20; found: 1017.94 [M+H]^+^, 545.39 [M+2H]^2+^; Figure S7. Fmoc-FFAAAKAA-NH_2_. Refer for legend of Figure S5 for chromatographic conditions. Purity 98.2%; Figure S8. Mass of Fmoc-FFAAAKAAA-NH_2_. Calculated: 1088.53; found: 1088.98 [M+H]^+^, 545.29 [M+2H]^2+^; Figure S9. Fmoc-FFAAAKAAA-NH_2_. Refer for legend of Figure S5 for chromatographic conditions. Purity 91.8%; Figure S10. FTIR Overlaid spectra of peptide EDP-2 supernatant after gelation (red) and Buffer-ACN solvent (blue); Figure S11. UV spectra of peptide EDP-2 before (black) and after gelation (red); Figure S12. Size distribution measurement of EDP-1. Figure S13. Size distribution measurement of EDP-2; Table S1. MolProbity computational data of EDP-1; Table S2. MolProbity computational data of EDP-2; Table S3. MolProbity computational data of EDP-3; Table S4. Rheology experiment of EDP-1; Table S5. Rheology experiment of EDP-2.
