# Supplementary material for: Elastin-Derived Peptide-Based Hydrogels as a Potential Drug Delivery System
Source: Gels. 2024 Aug 12;10(8):531. doi: 10.3390/gels10080531 (PMC11354093; doi:10.3390/gels10080531)
Supplement: Supplementary file 1 [file gels-10-00531-s001.zip › gels-3132949-supplementary.pdf]

gels

Supplementary Materials

Elastin-Derived Peptide-Based Hydrogels as a Potential Drug Delivery System

Supplementary Figures

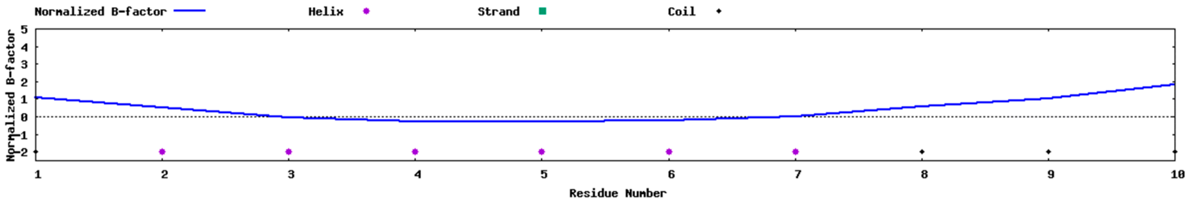

Figure S1. The normalized B-factor (called B-factor profile, BFP) of EDP-1.

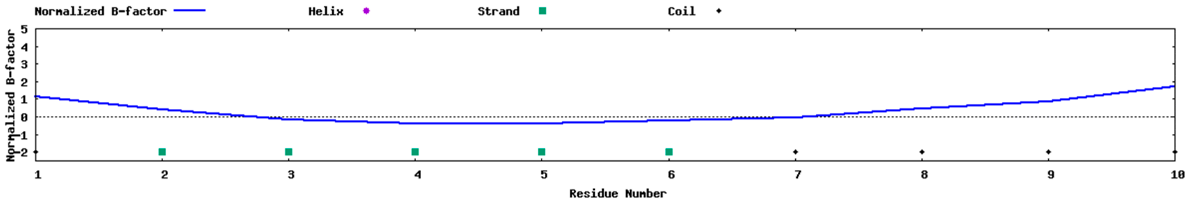

Figure S2. The normalized B-factor (called B-factor profile, BFP) of EDP-2.

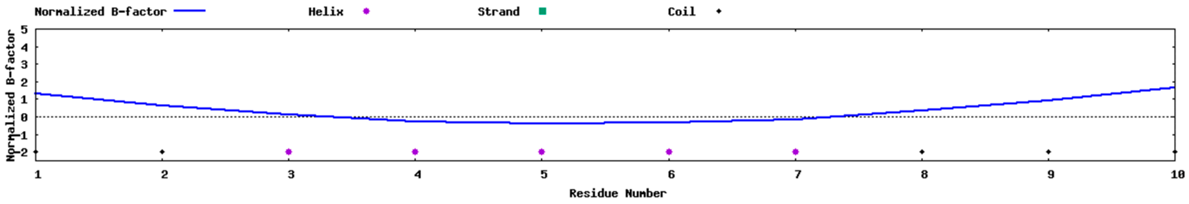

Figure S3. The normalized B-factor (called B-factor profile, BFP) of EDP-3.

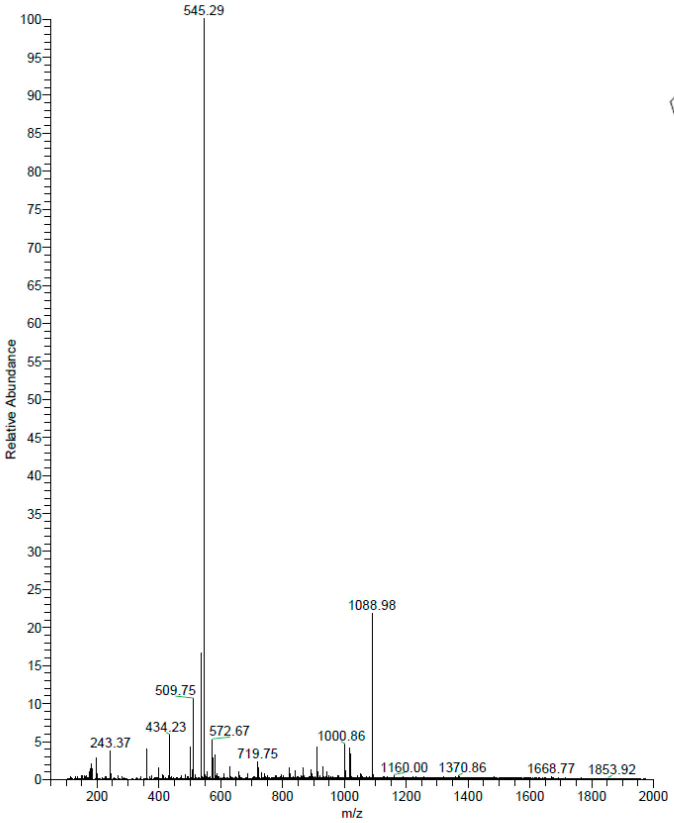

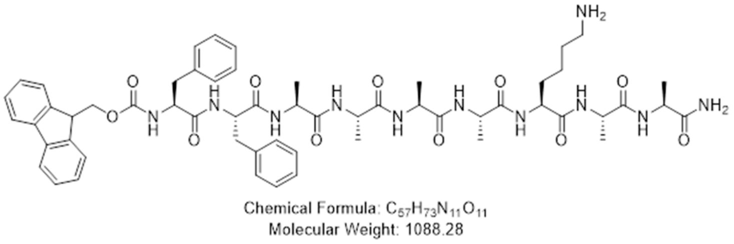

Figure S4. Mass of Fmoc-FFAAAAKAA-NH2. Calculated: 1088.28; found: 1088.98 $[M+H]^+$, 545.29 $[M+2H]^{2+}$.

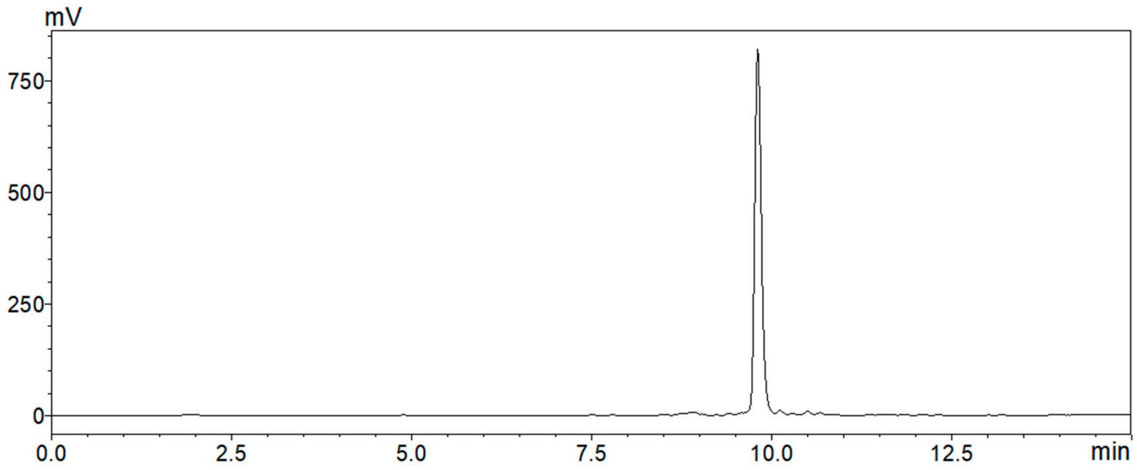

Figure S5. Fmoc-FFAAAAKAA-NH2. 5–95% in 30 min gradient elution. λ = 280 nm. Mobile phase A: 0.1% TFA in H_2O; mobile phase B: 0.1% TFA in CH_3CN; Symmetry Luna C18 (3.6 µm, 4.6 × 150 mm) column. Purity 98.2%.

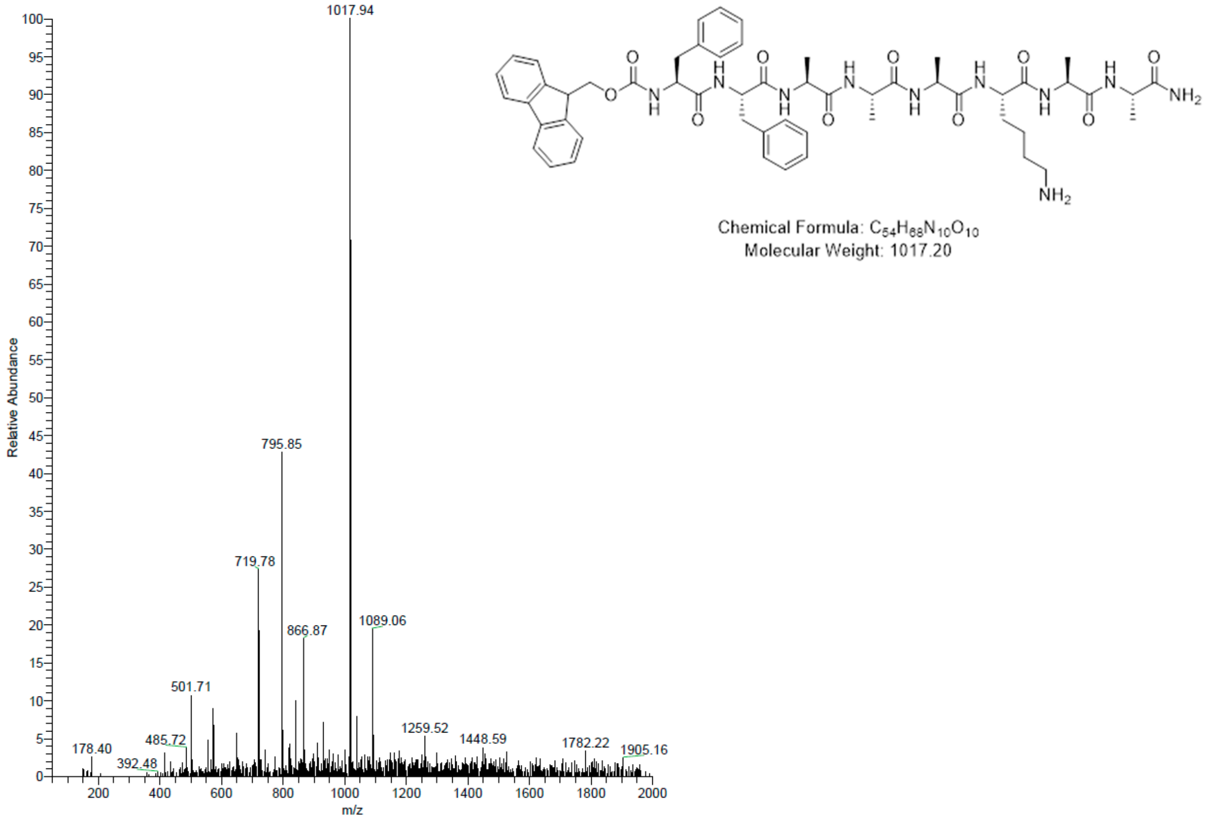

Figure S6. Mass of Fmoc-FFAAAKAA-NH_2. Calculated: 1017.20; found: 1017.94 $[M+H]^+$, 545.39 $[M+2H]^{2+}$.

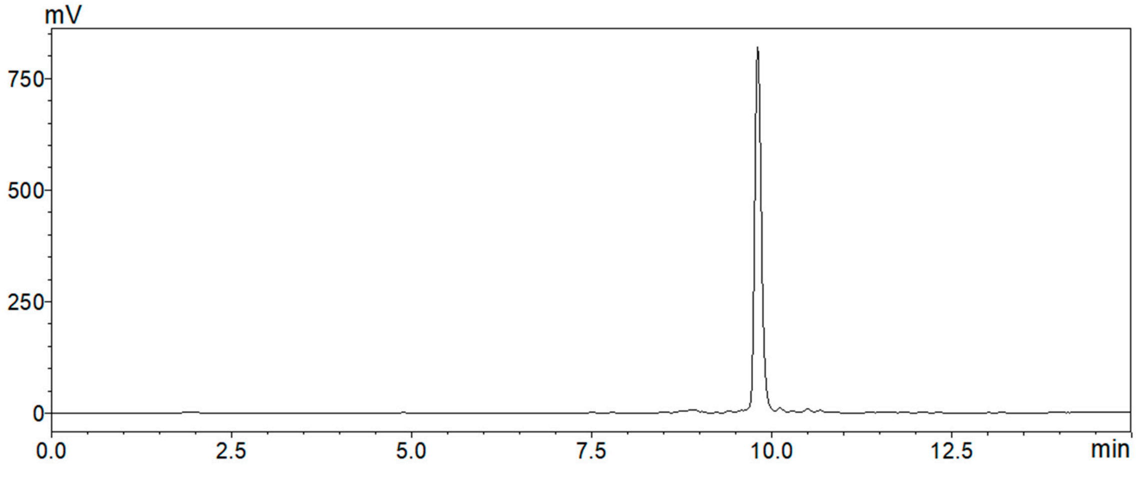

Figure S7. Fmoc-FFAAAKAA-NH_2. Refer for legend of Fig. S5 for chromatographic conditions. Purity 98.2%.

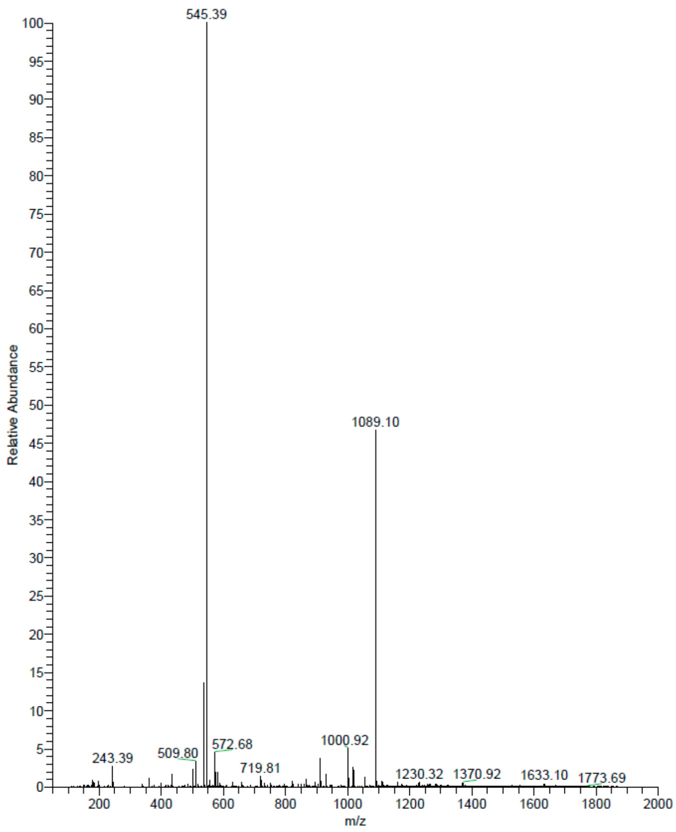

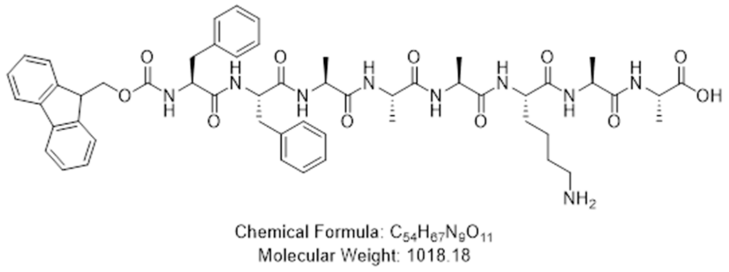

Figure S8. Mass of Fmoc-FFAAAKAAA-NH2. Calculated: 1088.53; found: 1088.98 $[M+H]^+$, 545.29 $[M+2H]^{2+}$.

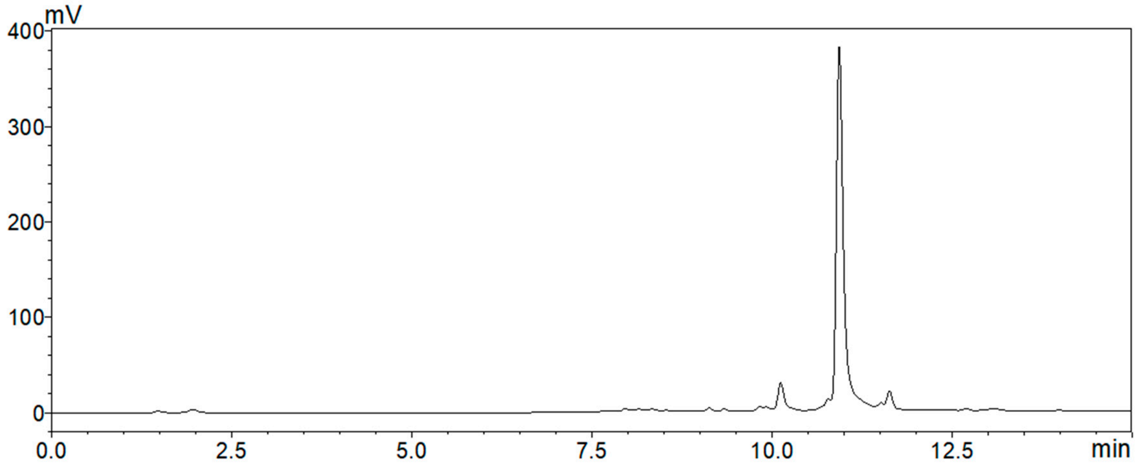

Figure S9. Fmoc-FFAAAKAAA-NH2. Refer for legend of Fig. S5 for chromatographic conditions. Purity 91.8%.

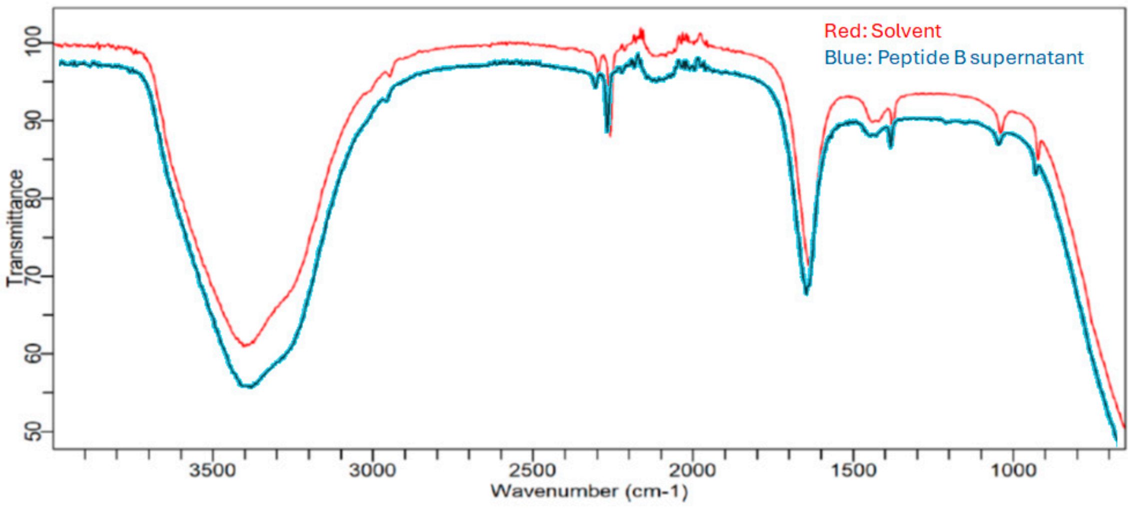

Figure S10. FTIR Overlaid spectra of peptide EDP-2 supernatant after gelation (red) and Buffer-ACN solvent (blue).

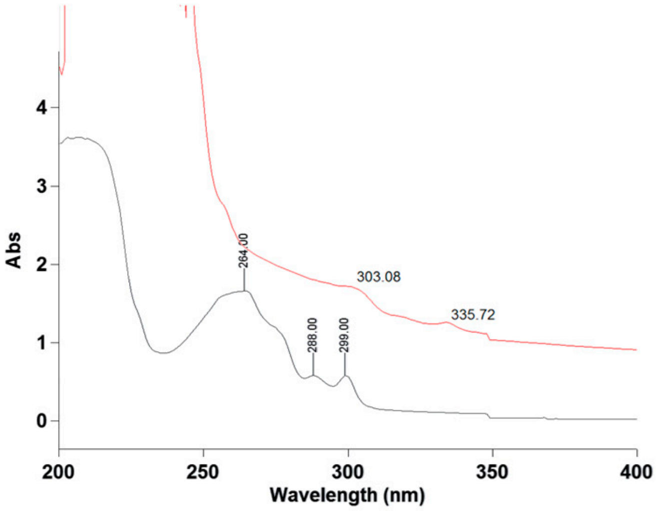

Figure S11. UV spectra of peptide EDP-2 before (black) and after gelation (red).

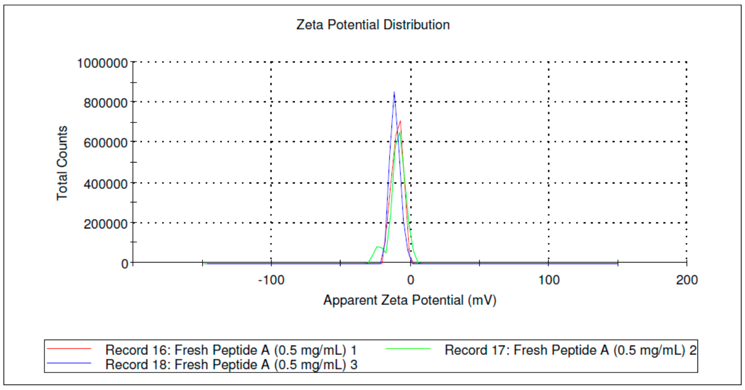

Figure S12. Size distribution measurement of EDP-1.

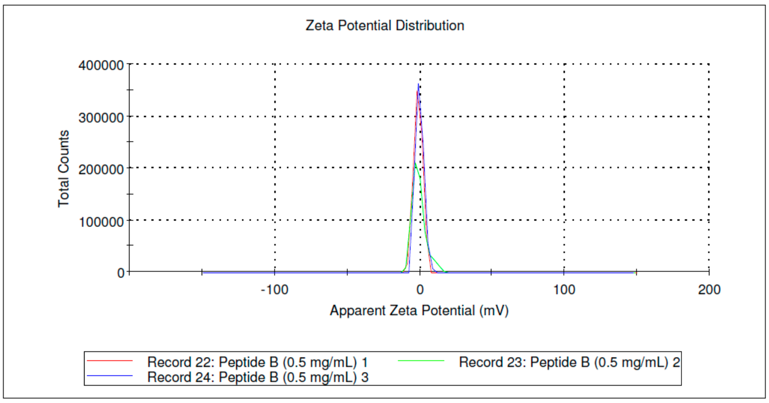

Figure S13. Size distribution measurement of EDP-2.

Supplementary Tables

Table S1. MolProbity computational data of EDP-1.

Residue	Type	Φ	Ψ	Z
F	Helix	-55.78	-30.39	-1.1045
F	Helix	-63.15	-45.58	1.4974
A	Helix	-65.62	-38.50	0.3733
A	Helix	-59.99	-45.70	0.4728
A	Helix	-57.47	-48.80	-0.5863
A	Helix	-62.51	-10.35	-1.2255
K	Helix	-109.96	-3.90	-1.1632
A	Helix	-129.42	-11.74	-1.2311

Table S2. MolProbity computational data of EDP-2.

Residue	Type	Φ	Ψ	Z
F	Loop	-64.68	164.49	-1.1246
F	Loop	-145.13	135.82	-0.5514
F	Helix	-143.86	124.93	-1.1566
A	Helix	-53.45	-49.93	-1.0691
A	Helix	-67.62	-26.80	-1.0792
A	Helix	-83.57	-5.18	-1.1831
K	Helix	-132.61	38.04	-1.1657
A	Helix	-65.51	38.92	-1.2315

Table S3. MolProbity computational data of EDP-3.

Residue	Type	Φ	Ψ	Z
F	Helix	-52.06	-38.91	-1.0464
F	Helix	-68.02	-34.76	-0.4102
A	Helix	-72.36	-43.91	-1.0751
A	Helix	-57.07	-50.58	-0.9270
A	Helix	-56.97	-46.79	-0.2311
K	Helix	-61.15	-32.12	-0.6923
A	Helix	-75.49	-17.54	-1.1827
A	Helix	-70.76	-10.35	-1.2040

Table S4. Rheology experiment of EDP-1.

Point Index	Shear modulus (elastic component) (Pa)	Shear modulus (viscous component) (Pa)

1	4.072	0.6968
2	4.298	0.7448
3	4.776	0.8298
4	5.276	0.8846
5	5.66	0.8938
6	6.206	0.9488
7	6.709	0.9976
8	7.392	1.121
9	7.956	1.191
10	8.631	1.258
11	9.476	1.362
12	10.29	1.543
13	11.51	2.191
14	11.96	1.718
15	13.17	1.887
16	14.31	2.089
17	15.17	2.233
18	16.7	2.403
19	18.1	2.651
20	19.63	2.946
21	21.28	3.191
22	22.93	3.375
23	24.55	3.797
24	26.18	4.017
25	27.96	4.22
26	30.01	4.664
27	31.87	4.93
28	33.74	5.301
29	35.73	5.545
30	37.62	6.033
31	39.74	6.386
32	40.82	6.288
33	43.2	6.827
34	44.82	7.081
35	46.87	7.696
36	48.81	8.077
37	50.98	8.752
38	53.04	9.167
39	55.12	9.621
40	57.13	10.08
41	59.08	10.84
42	54.97	14.36
43	54.36	9.851
44	58.24	10.41
45	60.41	10.6
46	62.5	11.42
47	63.92	12.25
48	65.01	12.65
49	65.86	13.42
50	66.6	13.54
51	66.99	13.94
52	67	14.27
53	67.18	14.33

54	66.96	14.41
55	66.48	14.64
56	66.42	14.45
57	67.21	13.35
58	68.69	12.78
59	67.21	13.17
60	69.06	13.18
61	69.13	14.36
62	69.79	14.68
63	70.74	13.32
64	58.9	14.32
65	65.27	13.14
66	67.2	13.52
67	65.23	13.25
68	67.11	14.27
69	67.78	15.02
70	67.04	14.22
71	67.72	14.92
72	69.08	15.21
73	71.22	15.44
74	73.34	14.84
75	75.33	14.82
76	77.28	15.41
77	77.85	16.37
78	78.7	16.99
79	79.6	17.04
80	76.96	16.66
81	80.24	16.17
82	83.05	15.76
83	83.31	18.67
84	81.51	18.7
85	84.06	19.91
86	83.42	19.88
87	85.15	21.15
88	83.19	20.04
89	86.66	21.13
90	88.99	18.73
91	91.83	17.73
92	93.12	18.46
93	91.3	20.65
94	79.56	23.22
95	91.79	22.36
96	93.55	22.71
97	95.36	22.96
98	97.37	22.9
99	98.51	23.68
100	100	23.7
101	94.88	38.11
102	100.1	23.71
103	101.9	24.46
104	101.3	24.25
105	104	24.4
106	103	28.52

107	101.8	23.62
108	105.5	22.56
109	107.8	22.9
110	109.4	23.64
111	109.6	25
112	110.4	26.29
113	107.8	25.02
114	107.6	29.14
115	109.9	27.03
116	111.6	27.97
117	115.3	26.78
118	117.2	27.68
119	118.2	29.16
120	117	29.09
121	117.3	34.93
122	119.6	29.05
123	120.9	28.55
124	115	36.76
125	123.1	28.32
126	125	30.71
127	100.8	41.81
128	124.2	30.79
129	122.1	31.09
130	125.5	31.61
131	128.1	32.73
132	127.3	35.12
133	129.5	33.32
134	132.5	34.27
135	135.6	44.18
136	124	28.67
137	123.2	29.09
138	126.3	31.44
139	131.4	33.94
140	128.1	31.51
141	132.5	34.75
142	136.5	35.93
143	135.8	34.33
144	135.7	34.29
145	138.6	36.42
146	135	35.07
147	141.9	37.03
148	145.2	38.34
149	139.2	35.03
150	145.8	37.95
151	148.2	38.04
152	150.1	38.83
153	153.7	40
154	156.7	41.08
155	158.4	42.12
156	157.3	43.89
157	160.6	41.71
158	163.8	43.2
159	165.3	44.09

160	167.8	45.06
161	168.7	48.59
162	165.5	42.11
163	169	44.88
164	171.6	45.84
165	174.9	46.65
166	168.8	44
167	174.8	46.12
168	178.5	45.25
169	173.6	47.67
170	178.1	44.45
171	180.8	45.43
172	171.6	40.67
173	170.1	51.71
174	176.8	44.17
175	182.7	44.78
176	186.7	46.08
177	183.3	44.88
178	185.6	46.91
179	187.7	47.41
180	191.1	47.6
181	192.6	48.84
182	194	48.38
183	195.8	47.44

Each point represents 1 min.

Table S5. Rheology experiment of EDP-2.

Point Index	Shear modulus (elastic component) (Pa)	Shear modulus (viscous component) (Pa)
1	7.999	1.314
2	8.971	1.467
3	10.05	1.697
4	8.622	1.947
5	7.661	3.01
6	10.89	1.94
7	11.96	2.302
8	13.95	2.615
9	14.98	2.888
10	17.16	3.42
11	19.17	3.839
12	21.4	4.451
13	21.01	4.195
14	24.53	5.242
15	27.79	6.113
16	31.65	7.376
17	34.79	8.28
18	39.83	9.699
19	44.36	11.85
20	50.4	13.39
21	56.01	15.99
22	62.24	18.29
23	69.07	20.99

24	75.44	24.06
25	81.33	26.88
26	60.53	18.34
27	76.18	17.01
28	70.5	16.4
29	63.93	16.12
30	60.24	16.13
31	64.5	14.87
32	67.36	16.55
33	67.98	17.08
34	72	16.77
35	74.94	16.17
36	78.42	16.65
37	81.04	16.62
38	83.04	16.62
39	83.93	17.27
40	61.61	15.12
41	70.64	17.31
42	73.92	17.85
43	77.75	19.72
44	86.7	20.13
45	89.67	19.81
46	88.44	21.12
47	76.55	20.76
48	77.24	22
49	77.62	20.65
50	82.45	21.96
51	83.07	23.43
52	77.96	22.32
53	63.66	24.49
54	85.05	23.94
55	89.14	27.95
56	94.5	27.72
57	99.25	28.1
58	68.09	25.9
59	90.71	28.18
60	90.92	26.24
61	97.44	27.87
62	88	20.68
63	100.3	30.21
64	105.4	32.27
65	112.2	33.44
66	118.5	37.27
67	109.2	37.71
68	106.6	33.05
69	96.02	32.26
70	105.2	34.48
71	114	42.07
72	112.6	37.85
73	120.5	39.46
74	133.2	50.46
75	105.2	46.57
76	119.2	49.25

77	117.9	41.75
78	67.48	16.92
79	118.2	36.17
80	127.6	43.66
81	129.5	48.2
82	138	67.61
83	110.6	50.58
84	129.5	52.39
85	138	54.96
86	148	59.04
87	161.6	62.4
88	140.8	74.07
89	147.3	59.32
90	170.7	69.82
91	190	84.74
92	201.5	76.42
93	207.8	79.29
94	216.4	89.6
95	237.5	87.71
96	215.1	119.1
97	219.2	91.02
98	228.9	99.58
99	197.6	128.1
100	95.65	135.3
101	222	105.5
102	233.2	106.5
103	259.8	124.1
104	275.2	131.2
105	335.9	146.8
106	365.6	132.3
107	364	143.5
108	349	132.1
109	48.69	117.3
110	306.4	160.9
111	329.8	173.4
112	364.2	64.81
113	352.1	189.9
114	365.6	203
115	35.52	133.5
116	413.3	209.7
117	419.5	175.8
118	449.4	255.4
119	497.6	277.5
120	474.4	327.7
121	510.8	292.7
122	562.1	348.3
123	606.9	380.2
124	685.1	410.4
125	703.1	299.2
126	429.4	284.1
127	837.4	454.7
128	830.2	523.6
129	923.8	571.5

130	995.2	628.5
131	964.9	692.6
132	1.20E+03	697.2
133	1.21E+03	688.6
134	1.29E+03	796.2
135	1.32E+03	833.5
136	1.43E+03	856.8
137	1.54E+03	899
138	1.55E+03	920.2
139	1.69E+03	937.8
140	1.78E+03	1.01E+03
141	1.81E+03	1.06E+03
142	130.5	547.3
143	1.85E+03	1.28E+03
144	2.14E+03	1.20E+03
145	2.28E+03	1.26E+03
146	2.28E+03	1.31E+03
147	2.30E+03	1.93E+03
148	2.46E+03	1.29E+03
149	2.69E+03	1.50E+03
150	2.87E+03	1.58E+03
151	2.99E+03	1.66E+03
152	3.09E+03	1.67E+03
153	3.19E+03	1.83E+03
154	2.17E+03	2.33E+03
155	3.46E+03	1.96E+03
156	3.74E+03	2.06E+03
157	3.94E+03	2.10E+03
158	4.08E+03	2.22E+03
159	4.44E+03	2.33E+03
160	4.40E+03	2.38E+03
161	4.59E+03	2.61E+03
162	4.80E+03	2.70E+03
163	4.92E+03	2.86E+03
164	5.15E+03	2.75E+03
165	5.51E+03	2.98E+03
166	5.67E+03	3.05E+03
167	5.91E+03	3.23E+03
168	6.10E+03	3.34E+03
169	6.33E+03	3.41E+03
170	6.30E+03	4.17E+03
171	6.93E+03	3.54E+03
172	6.72E+03	3.14E+03
173	6.84E+03	3.94E+03
174	6.11E+03	4.38E+03
175	7.52E+03	3.55E+03
176	7.58E+03	4.08E+03
177	8.36E+03	3.84E+03
178	7.79E+03	4.07E+03
179	7.88E+03	4.35E+03
180	8.24E+03	4.18E+03
181	8.33E+03	4.73E+03
182	8.51E+03	4.26E+03

183	4.13E+03	4.92E+03

Each point represents 1 min.
